# CrpH of *Bordetella pertussis*, a prototypic PepSY_TM protein supporting heme-copper oxidoreductase function

**DOI:** 10.3389/fmicb.2026.1786092

**Published:** 2026-03-18

**Authors:** Majda Hachmi, Gauthier Roy, Anne-Sophie Debrie, Stéphanie Slupek, Rudy Antoine, Françoise Jacob-Dubuisson

**Affiliations:** University of Lille, Inserm, CNRS, CHU Lille, Institut Pasteur de Lille, U1019-UMR9017-CIIL-Center for Infection and Immunity of Lille, Lille, France

**Keywords:** aerobic respiration, *Bordetella pertussis*, copper acquisition, heme-copper oxidoreductase, PEPSY_TM family, sequence similarity network, copper reductase

## Abstract

In the living world, copper is both toxic in excess and necessary for the activity of specific oxidoreductases and electron transfer chains and as such is involved in the host-pathogen interface. Mammalian hosts deploy anti-microbial strategies of copper intoxication or starvation of invading microorganisms, collectively called nutritional immunity, and bacteria have developed both protection and acquisition systems in response. We recently described a TonB-dependent copper importer, CrtA^Bp^ in the whooping cough agent *Bordetella pertussis*. Here we characterized another protein encoded in the same operon and similarly upregulated by copper starvation, CrpH. By combining *in vitro* and *in vivo* experiments with transcriptomics, we showed that CrpH contributes to bacterial fitness and enhances respiration by the heme-copper oxidoreductases (HCO) of *B. pertussis*. CrpH belongs to the PepSY_TM superfamily of membrane-associated bacterial enzymes, whose known members catalyze heme-mediated ferrisiderophore reduction in the periplasm. The corresponding heme-binding motifs of CrpH are similarly required for function. Furthermore, we uncovered a synthetic growth phenotype of a double *crpH*-*ccoG* mutant, the latter gene encoding a putative copper reductase involved in HCO assembly. *In silico* analyses identified thousands of CrpH orthologs, leading us to define a new subfamily of PepSY_TM proteins found in diverse bacterial species all harboring HCO genes. Collectively, our results indicate that CrpH of *B. pertussis* is a prototype of a family of proteins supporting HCO function.

## Introduction

A handful of transition metals are ubiquitously encountered in living beings, where these micronutrients play essential structural, regulatory and catalytic roles (Helmann, 2025a; Helmann, 2025b). However, metals are also toxic in excess, hence their homeostasis is strictly controlled. In particular, they are key players at the host-pathogen interface, with hosts deploying strategies to intoxicate invading microorganisms with metals or to withhold metals to starve them, collectively called nutritional immunity (Murdoch and Skaar, 2022). In turn, bacteria have developed a variety of systems to protect against toxic levels of metals or conversely to scavenge scarce metals from their environment (Chandrangsu et al., 2017; Fu et al., 2014).

Among transition metals, copper is an integral part of the catalytic center of several oxidative enzymes thanks to the redox potential of the Cu(I)/Cu(II) couple. Its properties have notably been harnessed in electron transport chains for energy generation (Andrei et al., 2020). Because copper is also toxic, bacteria have acquired several protection mechanisms. In contrast, relatively few copper acquisition systems are known in bacteria. A few species synthesize “chalkophores” to acquire copper (Buglino et al., 2022; Kenney and Rosenzweig, 2018; Koh et al., 2017). In diderm bacteria copper crosses the outer membrane through porins or TonB-dependent transporters (TBDTs), few of which are currently known to import this metal (Bhamidimarri et al., 2021; Hachmi et al., 2026). Specific copper uptake across the cytoplasmic membrane has mostly been described in respiratory heme-copper oxidoreductase (HCO) assembly pathways (Khalfaoui-Hassani et al., 2023; Khalfaoui-Hassani et al., 2018).

The whooping cough agent *Bordetella pertussis* harbors a large virulence regulon under the control of the two-component BvgAS system, but its metabolism also contributes to its success as a pathogen (Belcher et al., 2021). With respect to copper, *B. pertussis* has shed most protection systems against copper typically found in Proteobacteria, whereas it possesses a three-gene operon, *cruR-crtA-bp2921*, maximally expressed under conditions of copper limitation and involved in its acquisition (Hachmi et al., 2026; Rivera-Millot et al., 2021; Roy et al., 2022). The first gene codes for the copper-responsive upstream ORF CruR, which negatively regulates expression of the following two genes in a post-transcriptional manner in conditions of copper excess (Roy et al., 2022). The second gene encodes a TBDT, CrtA, that imports copper-chelate complexes for the HCOs of *B. pertussis* (Hachmi et al., 2026). HCOs are major cuproproteins in this strictly aerobic species which produces two such complexes, an aa3 cytochrome c oxidase (aa3-Cox) and a bo ubiquinol oxidase (bo-Qox), as well as a copper-independent bd ubiquinol oxidase (bd-Qox) (Kaila and Wikstrom, 2021; McKay et al., 2024). HCO assembly pathways have been characterized in a few model bacteria, although not in *B. pertussis* (Hederstedt, 2022).

The function of the protein encoded by *bp2921*, the last gene of the *cruR-crtA-bp2921* operon, remains unknown. Because the functions of both CruR and CrtA are linked with copper, we hypothesized that the third protein of the operon may also be copper-related. This protein (Uniprot Q7VUZ4) belongs to the Pfam PepSY_TM family, widespread among eubacteria. PepSY_TM proteins harbor up to six transmembrane helices with nested, loosely conserved 60 to 90 residue-long extracytoplasmic *α*/*β* PepSY domain(s). A few of them have been characterized as being periplasmic ferrisiderophore reductases, thereby promoting dissociation of iron for its uptake across the cytoplasmic membrane (Hussein et al., 2024; Josts et al., 2021; Ong and O’Brian, 2023). The crystal structure of a family member, FoxB of *Pseudomonas aeruginosa*, shows a large periplasmic moiety formed of two PepSY domains, each flanked by two transmembrane helices. Highly conserved His residues on each side of the membrane axially chelate two heme groups for electron transfer from an unknown donor to the ferrisiderophore bound in the periplasmic cavity of the enzyme (Josts et al., 2021).

In this work, we investigated the function of the protein encoded by *bp2921*. Because CrtA was shown to import copper for the HCOs of *B. pertussis*, we tested the hypothesis that the *bp2921*-encoded protein also contributes to HCO-mediated respiration. We found that its activity enhances the *in vitro* and *in vivo* fitness of *B. pertussis*. Our results indicate that it contributes to HCO function possibly as a periplasmic copper reductase, hence the proposed name CrpH (copper reduction in the periplasm for HCOs). *In silico* analyses identified thousands of putative orthologs in various bacterial genera, suggesting that CrpH is the prototype of a new subfamily of PepSY_TM proteins functionally associated with HCO-mediated respiration.

## Materials and methods

### Strains and plasmids

Several *crpH* mutant strains were constructed in this study. An in-frame deletion was generated by amplifying the flanking regions of *bp2921* and cloning them into pSS1129 (Stibitz, 1994). The recombinant plasmid was introduced into *E. coli* SM10 and transferred to *B. pertussis* BPSM by conjugation for allelic exchange, yielding Bp*-*Δ*crpH*. We also constructed a *crpH* KO mutant (Bp*-crpH*_KO_) by insertion of the suicide plasmid pFUS2 (Antoine et al., 2000). To compare growth profiles between strains harboring the same resistance markers, a gentamycin-resistant BPSM variant was used where necessary (Solans et al., 2018). Site-directed mutagenesis was used to introduce the H_200_A and H_444_A substitutions in *crpH*, followed by allelic exchange to reintroduce the gene in Bp*-*Δ*crpH*. Knockout mutants of *cydAB* (*bp259-260*), *cyoABCD* (*bp2930*-*bp2933*), *ctaCDFGE* (*bp3740-bp3744*), and *ccoG* (*bp2173*) were obtained by interrupting the first gene of each operon with pFUS2. For heterologous expression, *crpH* was PCR-amplified and inserted into NdeI- and HindIII- restricted pET-TEV, yielding pET-TEV2921 which encodes CrpH with an N-terminal 6-His tag. The NcoI-HindIII fragment of the latter plasmid was also introduced in pBAD-mycHis (Invitrogen) for expression under the Para promoter. To express *crpH* under the pT5-lac promoter, the sequence containing the ribosome-binding site, the 6-His tag and the 5′ portion of *crpH* sequence was PCR-amplified from pET-TEV2921, restricted with BglII and ApaI fragment, and inserted in a three-piece ligation with the ApaI-HindIII fragment from the same plasmid in pCA24 (Kitagawa et al., 2005). For overexpression in *B. pertussis*, we introduced the P_120_A + P_121_A substitutions in *cruR* by allelic exchange in the *B. pertussis* chromosome, as they deregulated and markedly increased expression of a reporter gene replacing *crtA* and *crpH* (Roy et al., 2022). However, the resulting clones failed to produce CrtA, indicating selective pressure against increased expression of the operon. All PCR-based constructs were verified by Sanger sequencing to confirm the absence of extraneous mutations. Strains, plasmids, and oligonucleotides used in this study are listed in Supplementary Table S1.

### Culture conditions

*B. pertussis* was cultivated on Bordet–Gengou (BG) agar supplemented with defibrinated sheep blood for 48 h at 37 °C. Bacterial precultures were carried out in sterile plastic flasks to minimize metal contamination in Stainer–Scholte (SS) liquid medium (initial OD_600_ = 0.25) and incubated for 20 h at 37 °C with shaking. For high-aeration conditions, cultures were inoculated from these precultures into 96-well plates (200 μL of medium per well) at an initial OD_600_ = 0.15 and grown at 37 °C under continuous agitation (400 rpm) in the EnSight Multimode Plate Reader (PerkinElmer). For low-aeration conditions, a second preculture was performed in SS medium supplemented with 500 μM Trien, except with the *cydAB* mutants for which the second preculture was performed without Trien. Static cultures were inoculated in covered 96-well plates (200 μL of medium per 390-μL flat-bottom well) at initial OD_600_ = 0.2. Plates were briefly agitated for 5 min every hour to homogenize bacterial suspensions (400 rpm, orbital diameter of 1 mm) followed by 1 min without agitation prior to OD measurements. For all growth assays, SS medium was supplemented with 20 mM MgSO_4_ to maximize *crpH* expression, and ascorbic acid was omitted from the SS medium to minimize Cu reduction, except where indicated. For copper supplementation, CuSO_4_ was added at the indicated concentrations. Antibiotics were used at final concentrations of 100 μg/mL streptomycin and 10 μg/mL gentamycin for strains carrying pFUS2. For recombinant expression of *crpH*, various media, temperature and induction conditions were tested with all plasmids. pET-TEV2921 was introduced in *E. coli* C43(DE3) and grown in LB or terrific broth at 37 °C, for 4 h with 1 mM IPTG or 20 °C overnight with 100 μM IPTG; or in auto-induction medium (AIM) at 37 °C for 4 h then 20 °C overnight. For expression in pBAD, *E. coli* LMG194 was grown in LB or terrific broth medium for 4 h at 37 °C then 16 °C overnight with 0.2% arabinose. For pCA24-2921, expression was performed in *E. coli* UT5600 or BL21 grown in M9 medium at 18 °C overnight with 200 μM or 1 mM IPTG; or in AIM at 28 °C; or in LB for 4 h at 37 °C then overnight at 16° C with 200 μM or 1 mM IPTG. In all conditions 250 μM NH_4_Fe(SO_4_)_3_ and 50 μM levulinate were added.

### ATP quantification

Intracellular ATP levels were quantified using the BacTiter-Glo^™^ Microbial Cell Viability Assay (Promega). Bacteria were grown either under agitated or static conditions in 96-well plates containing 200 μL of medium per well supplemented with 2 μM CuSO_4_. After 24 h for agitated cultures and 24 h or 48 h for static cultures, 100 μL was used to measure the OD_600_, and the remaining 100 μL was mixed with an equal volume of reagent in white opaque 96-well plates. After 5 min of incubation at room temperature, light emission was measured using a Tecan microplate reader. Four biological replicates, each with three technical replicates were performed, and the results were expressed as relative light units (RLU) normalized to the OD_600_.

### TMPD assay

Bacteria grown in T25 flasks (10-mL cultures) were harvested at early or late-exponential growth phase and during late-exponential growth phase for high- and low-aeration conditions, respectively. They were diluted in 50 mM Na-phosphate buffer (pH 7.0), 150 mM NaCl, mixed with 0.5 mM TMPD (from a 10 mM stock solution in the same buffer containing 100 μM ascorbate), and TMPD oxidation was measured by recording absorbance at 562 nm using a Uvikon spectrophotometer. Initial rates of reaction were used to calculate the enzymatic activities, which were normalized to the OD_600_ value of the corresponding culture. Cytochrome c oxidase activity measured for the aa3-Cox-deficient Bp-*ctaCDFGE*_KO_ mutant, corresponding to non-specific oxidation, was subtracted from the values measured for the other strains.

### RNA extraction and sequencing

The bacteria were grown in T25 flasks with or without agitation (200 rpm) in SS medium supplemented with 20 mM MgSO_4_. Bacterial suspensions (8 mL) were collected at mid-exponential phase and centrifuged at 4,000 rpm for 15 min at 4 °C after adding 2 mL of a phenol:ethanol solution (5:95, v/v). Total RNA was extracted using Tri-Reagent (Invitrogen), followed by two consecutive DNase I treatments (Sigma-Aldrich), each followed by purification using AMPure XP beads (Beckman Coulter) to remove genomic DNA and contaminating solvents. RNA concentration was measured with a NanoDrop spectrophotometer (Thermo Fisher Scientific), and RNA integrity was assessed using an Agilent 2100 Bioanalyzer with the RNA 6000 Nano kit (Agilent Technologies). Ribosomal RNA was depleted using the QIAseq FastSelect-5S/16S/23S Kit (Qiagen). Strand-specific RNA-seq libraries were prepared with the QIAseq Stranded RNA Library Kit (Qiagen) according to the manufacturer’s instructions. Libraries were sequenced on an Illumina NextSeq 500 system (Illumina, Inc.) in high-output mode. All samples were multiplexed on one lane of the flow cell and sequenced in single-read sequencing mode with read lengths of 150 base pairs. Raw RNA-seq reads were processed with Illumina quality control tools using default settings. The data were deposited in ArrayExpress,^1^ under the accession number E-MTAB-16727.

### RNA-seq data analyses and dataset filtration

Raw sequencing data from three biological replicates of the two strains in each growth condition were processed using the SPARTA pipeline (Johnson et al., 2016). Briefly, the reads were first processed with Trimmomatic to remove Illumina adapter sequences and low quality reads with the following parameters (adapters/truseq3-se.fa:2:30:10; leading:3; trailing:3; slidingwindow:4:15; minlen:36). The quality of the reads was then assessed using fastQC. They were mapped on the *B. pertussis* TohamaI reference genome using Bowtie. The number of reads mapped to the different features was calculated using HTSeq-count. Transposase-encoding genes and pseudogenes were removed from the output tables containing reads per kb and million bp (RPKM) values for all annotated genes. The differential gene expression analysis for all the other genes was done using the edgeR package. Potential Batch Effects were detected using MultiDimensional Scaling (MDS) plot. Differential expression thresholds were set at log_2_FC ≥1 for upregulation and log_2_FC ≤−1 for downregulation, with an FDR ≤0.05 considered statistically significant. The results were visualized as a volcano plot generated with GraphPad Prism. A functional enrichment analysis (over-representation analysis) was performed on the lists of genes specifically up- or down-regulated in Bp-Δ*crpH* using the R programming language. Genes were first mapped to the STRING database using the *B. pertussis* taxon (TaxID 257,313) to assign a unique STRING identifier to each gene. Genes that could not be mapped were excluded from subsequent analyses. The remaining genes were associated with functional terms using all proteins annotated in STRING as the reference background. Functional enrichment was performed using the clusterProfiler R package with a *p*-value ≤0.05 adjusted using the Benjamini–Hochberg correction. For each functional term, a fold-enrichment value was calculated as the ratio between the numbers of genes associated with a given term in the list of interest and in the reference background. This metric was used to evaluate the degree of over-representation of each functional term in the analyzed gene set. The main enriched functional categories were visualized graphically according to fold enrichment, adjusted *p*-value, and the numbers of genes.

### Animal experiments

BPSM harboring a gentamycin resistance marker (Solans et al., 2018) and Bp-Δ*crpH* cultivated in standard SS medium were used to inoculate agitated cultures under copper-limiting conditions in medium with 50 μM of bathocuproine disulfonate (BCS) to maximize expression of the operon. Bacteria were diluted in sterile PBS, and mice were infected via the intranasal route with 5 × 10^4^ CFU in a 20-μL inoculum. For co-infection experiments, mice received an equal mixture of BPSM and Bp-Δ*crpH*, each at 5 × 10^4^ CFU per mouse. Groups of three or four mice for BPSM and four for Bp-Δ*crpH* were anesthetized using euthasol (140 mg/kg) followed by cervical dislocation after 3 h, 4 days, 7 days, and 14 days post-infection. Lungs and nasal cavities were collected, homogenized in PBS, and dilutions were plated on BG agar to determine CFU counts for each strain. In co-infection experiments, the two strains were differentiated by their antibiotic resistance markers. The study protocol was approved by the Ethical Committees of the Region Nord-Pas-de-Calais and the Ministry of Research under the agreement number APAFIS #51236-2024041114331031 v7.

### Statistics

Statistical analyses were performed using a nonparametric permutation-based ANOVA with a global model including two factors and their interaction (strain × time for animal experiments; strain × culture condition for TMPD and BacTiter-Glo assays). Significance was assessed using 10,000 permutations. When a significant interaction effect was detected, strain comparisons were performed independently at each time point or condition using permutation-based one-way ANOVA. Resulting *p*-values were adjusted for multiple testing using the Benjamini–Hochberg procedure. Effect sizes and confidence intervals were calculated according to Cohen’s *d* test.

### *In silico* analyses

All PepSY_TM proteins were first collected from the nr NCBI database (1/1/2025) using the PepSY_TM.hmm profile,^2^ sequence redundancy was reduced to 80% identity using CD-HIT,^3^ and the proteins (>40,000) were subjected to sequence similarity network analyses (SSN) using the Enzyme Function Initiative Enzyme Similarity Tool (EFI-EST)^4^ (Oberg et al., 2023). Networks were visualized by Cytoscape (Shannon et al., 2003). To build a weblogo (Crooks et al., 2004), we aligned 63 orthologs with ClustalW. ConSurf was used to analyze the conservation in this set. Co-occurrences analyses were performed on fully assembled bacterial genomes (>40,000) as described (Antoine et al., 2025). BlastP analyses were performed with all PepSY_TM proteins encoded in these genomes, and those whose first hit belongs to the CrpH SSN cluster were collected and considered CrpH orthologs.

### Immunoblot analyses

Antibodies against a synthetic CrpH peptide were produced in rats (Eurogentec) and used at a 1:500 dilution for detection of the protein from *B. pertussis* extracts, followed with an anti-rat-HRP conjugate (dilution 1:5,000; Promega). The recombinant protein produced in *E. coli* was detected using an antibody against a His6 tag (SigmaAldrich) followed with an anti-mouse–HRP (dilution 1:5,000; Promega). Detection was performed with the Amersham ECL Prime Western Blotting System using the Amersham Imager 600 (GE).

## Results

### Conditional growth defect of the *crpH* KO strain

**Figure 1:**
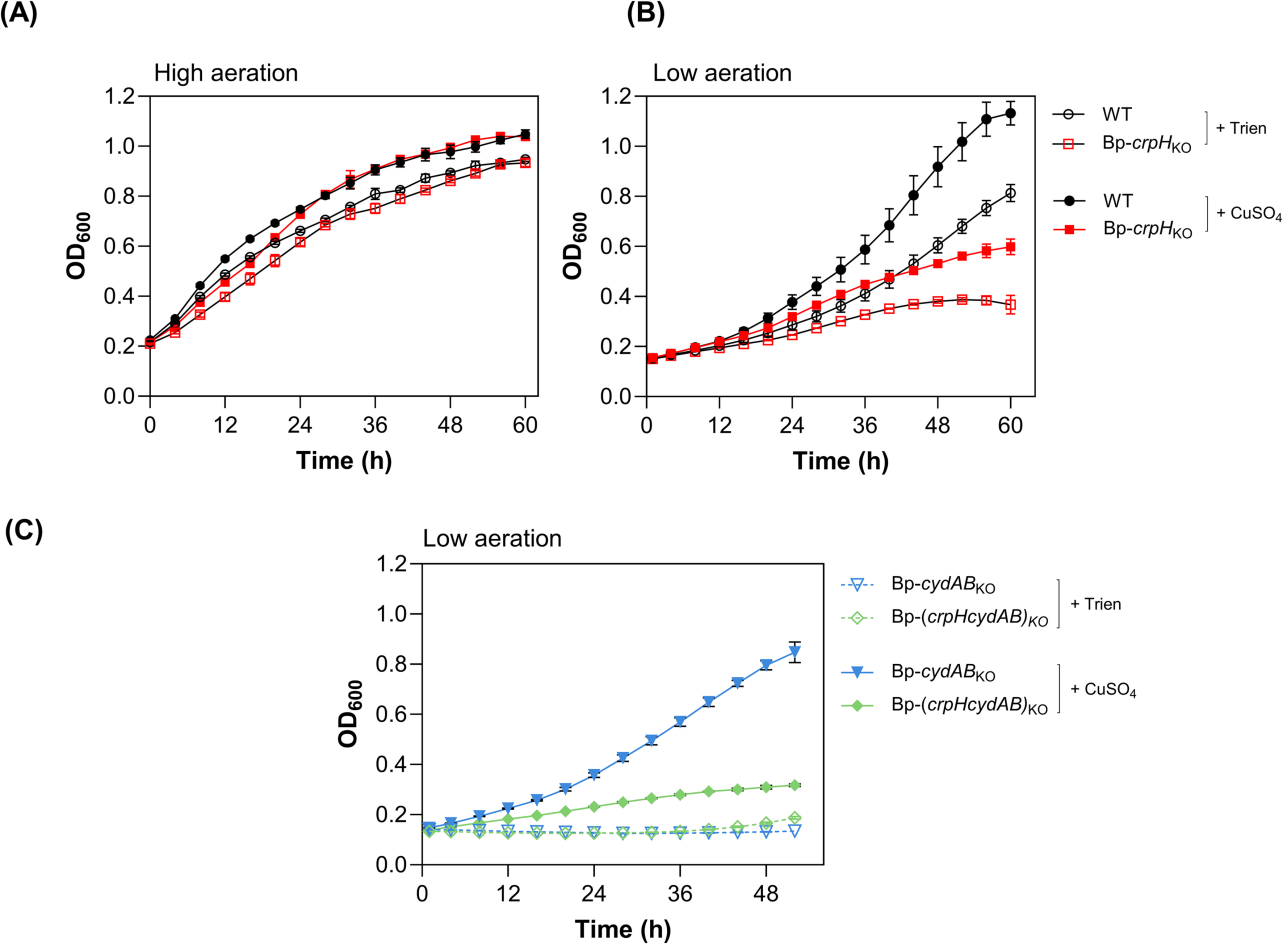
Growth phenotypes of the *crpH* mutant. **(A,B)** The growth of Bp-*crpH*_KO_ was compared to that of the WT strain under high- **(A)** or low-aeration **(B)** conditions. The strains were cultured under copper-limiting conditions (500 μM Trien) or in medium supplemented with CuSO_4_ at 2 μM in 96-well plates. **(C)** Bp-*cydAB*_KO_ and Bp-(*crpHcydAB*)_KO_ were grown under low-aeration conditions in standard medium supplemented with 500 μM Trien or 10 μM CuSO_4_. Representative growth curves from three independent biological replicates are shown. The data represent the means ± SD of three technical replicates.

To determine the role of CrpH in *B. pertussis*, we inactivated the corresponding gene by insertion. The growth of the resulting Bp-*crpH*_KO_ strain was compared to that of its wild-type (WT) parent, BPSM, in medium supplemented with the specific Cu(II) chelator triethylenetetramine (Trien) in 96-well plates under standard high-aeration culture conditions. Trien was added to limit copper availability and thereby to maximize *crpH* expression. Under these conditions however, Bp-*crpH*_KO_ exhibited no growth defect relative to its parent, and addition of CuSO_4_ hardly improved growth of the two strains (Figure 1A).

In contrast, when cultured under static, low-aeration conditions in copper-limited medium, Bp-*crpH*_KO_ exhibited a pronounced growth defect relative to its WT parent (Figure 1B). In copper-supplemented medium, both strains reached higher cellular densities, implying that this metal is key to facing low aeration. However, growth of the mutant strain remained much lower than that of the WT parent, even at a high copper concentration (Figure 1B; Supplementary Figure S1). Thus, optimal *B. pertussis* growth under low-aeration conditions requires both copper and *crpH*. The conditional growth defect of the mutant in low aeration hints at a role of CrpH in respiration.

Attempts to complement the deletion mutant were unsuccessful, most likely because the complex regulation of the operon could not be reproduced in trans (Roy et al., 2022). Because *crpH* is the last gene of the operon and the adjacent *bp2920* gene is transcribed in opposite orientation, a polar effect of *crp*H inactivation was very unlikely. We nevertheless confirmed the phenotype observed with Bp-*crpH*_KO_ by introducing an in-frame deletion in *crpH*. Growth of the resulting Bp-Δ*crpH* mutant in low-aeration conditions was similar to that of Bp-*crpH*_KO_, confirming that the conditional growth phenotype was caused by *crpH* inactivation (Supplementary Figure S2).

### Importance of CrpH for HCO-mediated respiration

To substantiate a potential role of CrpH in HCO-dependent respiration, the *cydAB* operon coding for the copper-independent bd-Qox was inactivated in both Bp-Δ*crpH* and the parental strain, yielding Bp-(*crpHcydAB*)_KO_ and Bp-*cydAB*_KO_, respectively. In the absence of *cydAB*, *B. pertussis* growth should become strictly dependent on copper, which is required for HCO function. Growth of Bp-*cydAB*_KO_ and Bp-(*crpHcydAB*)_KO_ was monitored under low-aeration conditions in copper-limiting medium or in medium supplemented with CuSO_4_. As expected, the growth of both strains was impaired in copper-limiting medium. CuSO_4_ supplementation markedly improved the growth of Bp-*cydAB*_KO_, whereas that of Bp-(*crpHcydAB*)_KO_ remained modest (Figure 1C). The limited growth rate of Bp-(*crpHcydAB*)_KO_ under low-aeration conditions even with copper supplementation suggested a defect in using copper for respiration in the absence of CrpH, consistent with the idea that CrpH somehow sustains the activity of one or both HCO(s) in *B. pertussis*.

**Figure 2:**
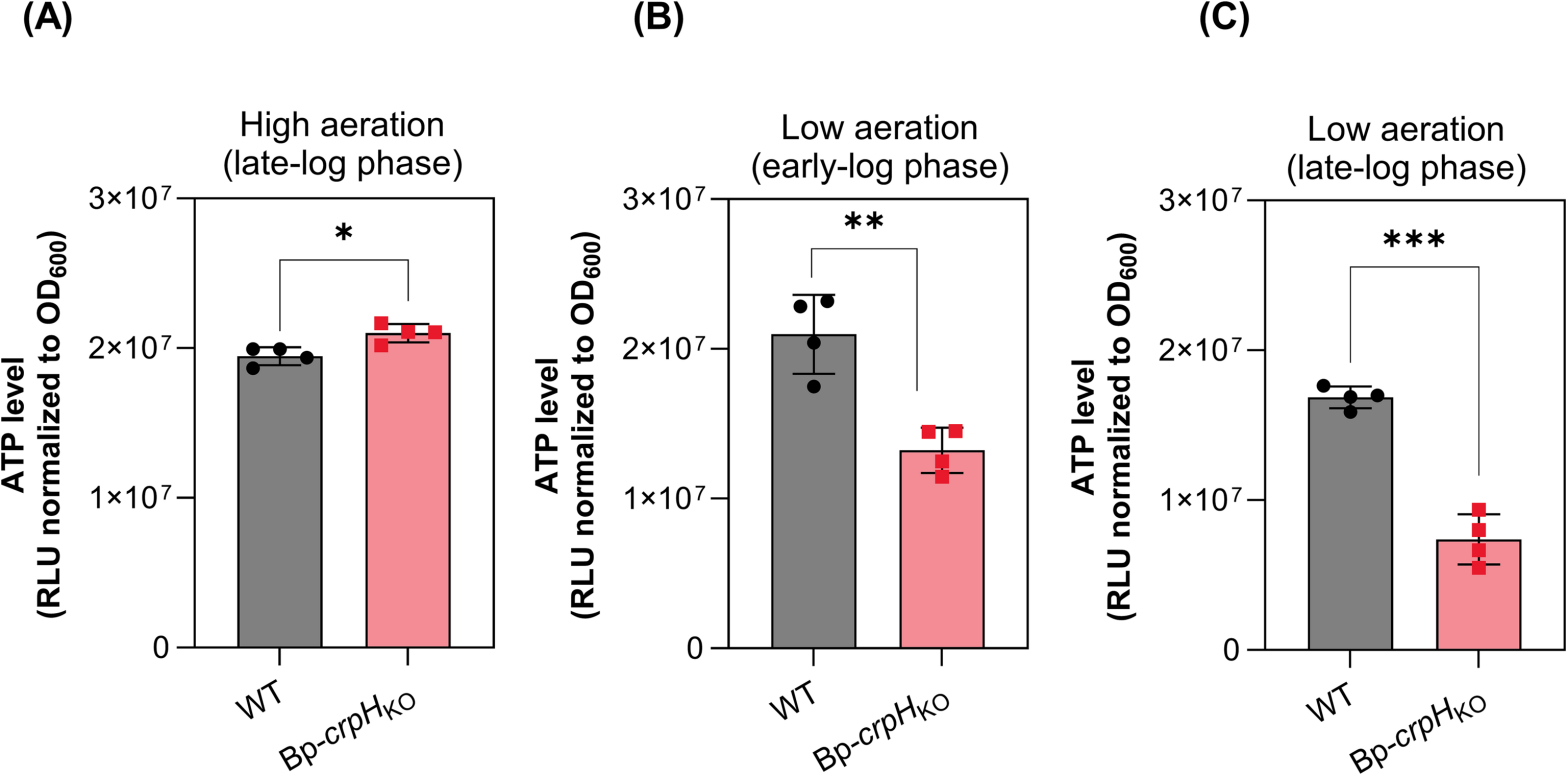
ATP levels in the *crpH* mutant relative to the WT strain. ATP levels were measured using the BacTiter-Glo assay. WT and *Bp-crpH*_KO_ were cultivated under high- **(A)** or low-aeration conditions **(B,C)** in medium supplemented with 2 μM CuSO_4_. Luminescence values are expressed as relative light units (RLU) normalized to the optical density of the cultures at 600 nm. Data represent the mean ± SD of four independent biological replicates. Statistical significance was determined using a non-parametric permutation-based ANOVA (^*^p < 0.05, ^**^p < 0.01, and ^***^p < 0.001).

We compared the intracellular ATP levels in the WT and Bp-*crpH_KO_* strains grown with CuSO_4_. In high-aeration conditions, ATP levels did not markedly differ between the two strains (Figure 2A). In contrast, they were reduced in Bp-*crpH_KO_* relative to its parent in low-aeration conditions both in early- and late-log phases, supporting a role of CrpH in respiration efficiency (Figures 2B,C).

We next inactivated *cyoABCD* or *ctaCDFGE*, coding for bo-Qox and aa3-Cox respectively, in Bp*-*Δ*crpH*. However, the resulting mutant strains hardly grew at all in low-aeration conditions, while the same KO mutations had milder effects in the parental strain. In contrast, the growth rates of these mutants were hardly reduced relative to the WT parent in high-aeration conditions (Supplementary Figure S3). Collectively, these results might be interpreted as follows. In high-aeration conditions, the copper-independent bd-Qox might be a major respiratory oxidase for *B. pertussis*, accounting for the absence of phenotypes of the *crpH* mutant. In low-aeration conditions in contrast, optimal growth requires HCO activity, and the role of CrpH becomes apparent. Thus, our results are consistent with a role for CrpH in handling respiratory copper.

**Figure 3:**
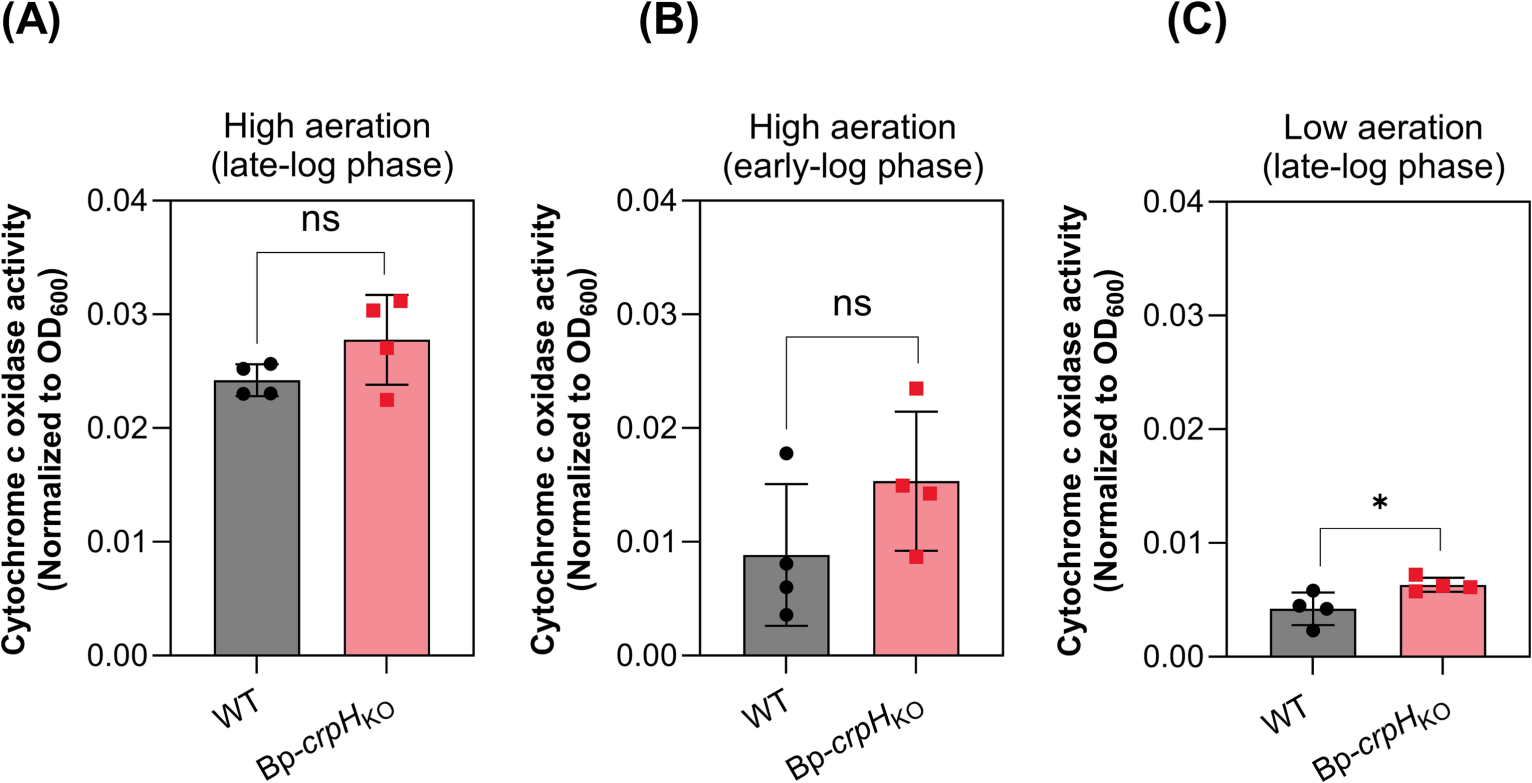
Cytochrome c oxidase activity in the crpH mutant relative to the WT strain. WT and *Bp-crpH*_KO_ were grown under high-aeration conditions to late-log phase in the presence of 2 μM CuSO_4_ **(A)**. To assess if aa_3_-Cox accumulation over the duration of the culture or the presence of CuSO_4_ might mask the effect of crpH inactivation, the activity was also measured in early-log phase without added copper in high-aeration conditions **(B)**, and in late-log phase without added copper in low-aeration conditions **(C)**. The cytochrome c oxidase activities were normalized to the optical density of the cultures at 600 nm. Data are shown as mean ± SD of four biological replicates. Statistical significance was determined using a non-parametric permutation-based ANOVA (^*^p < 0.05).

The activity of cytochrome c oxidases can easily be measured in bacteria using the chromogenic molecule TMPD, which turns blue when oxidized. We thus determined aa3-Cox activities in the WT and Bp-*crpH*_KO_ strains by spectrophotometry. Both strains exhibited similar activities both in high- and low-aeration conditions even without CuSO_4_ addition (Figure 3). Therefore, these results suggest that CrpH is not essential for aa3-Cox activity, although this rather crude assay might overlook moderate differences. Unfortunately, we could not measure bo-Qox activity, as no simple assay is available for ubiquinol oxidase activity. Therefore, these data did not enable us to unequivocally identify the potential client(s) of CrpH activity.

### Effect of *crpH* inactivation on gene expression

To investigate the defect of Bp-Δ*crpH*, we compared its transcriptomes under high- or low-aeration conditions with RNA-seq, using the transcriptomes of the WT strain in the same conditions as references (Supplementary Table S2). The culture conditions markedly affected gene expression in both strains, and the mutant displayed a larger number of differentially expressed genes than the WT strain, indicating that *crpH* inactivation impacts the *B. pertussis* transcriptome.

**Figure 4:**
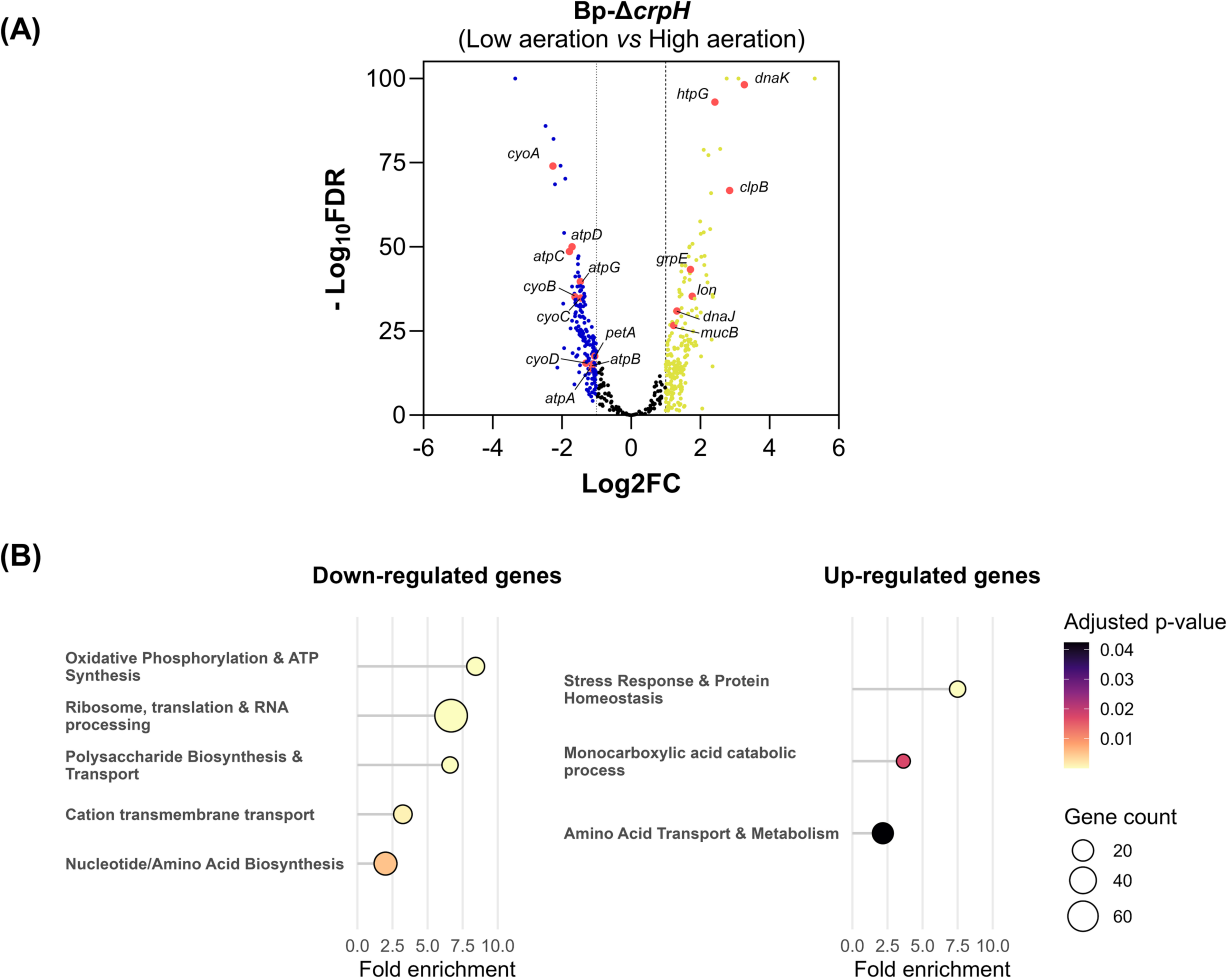
Differentially expressed genes in the *crpH* mutant under low- versus high aeration conditions. **(A)** Volcano plot of the genes of interest, with the log_2_fold change (FC) plotted on the *x*-axis and the statistical significance (−log_10_ FDR) on the *y*-axis. Upregulated and downregulated genes are shown in yellow and blue, respectively. Selected genes of interest are labeled on the plot. Genes with log_2_FC ≥1 or log_2_FC ≤−1 and FDR ≤0.05 were considered significantly differentially expressed. Data represent the mean of three independent biological replicates. **(B)** Over-representation analysis of genes specifically down- or upregulated in the Bp-ΔcrpH strain in low-aeration conditions. Enriched genes were grouped into functional categories, with the fold enrichment indicating the magnitude of over-representation in the functional categories. The circle sizes indicate the number of genes associated with each functional category, and the colors represent the adjusted *p*-values (*p* ≤ 0.05). The full data set is available in Supplementary Table S2.

We found 214 genes expressed at lower level (log₂FC ≤−1) under low-aeration conditions in Bp-Δ*crpH* compared to 104 in the WT strain, and 390 genes expressed at higher levels (log₂FC ≥1) under low-aeration conditions in the mutant strain compared to 243 in its parent. After removing genes that vary in the same direction in both strains and thus reflect culture conditions, we analyzed the genes with a specific regulation status in the mutant in low-aeration conditions (Figure 4A). Among these, *cyoA*, *cyoB*, *cyoC* and *cyoD* encoding the bo-Qox complex, and *atpA*, *atpB*, *atpC*, *atpG*, and *atpD*, encoding subunits of the ATP synthase complex, were downregulated in the mutant only. Conversely, several chaperone and protease genes (*dnaK*, *dnaJ*, *hptG*, *clpB*, *grpE*, *lon*, *mucD*) were up-regulated. An over-representation analysis was performed to define the major functional pathways impacted by *crpH* inactivation (Figure 4B). Genes coding for ribosomal components, translation and transcription, polysaccharide, nucleotide and amino acid biosynthesis and transport were enriched among the down-regulated genes, consistent with a slow-down of *B. pertussis* metabolism and growth. Among the upregulated genes, we found genes involved in the stress response as well as genes for the transport and catabolism of amino acids. Thus, according to this analysis, the absence of CrpH affects the metabolism of *B. pertussis* and generates stress in low-aeration conditions. The impact on *cyoABCD* expression suggests that inactivation of *crpH* affects the regulation of respiratory pathways, further supporting a role of CrpH for HCO activity, possibly on bo-Qox.

### CrpH contributes to the fitness of *Bordetella pertussis* in the murine respiratory tract

**Figure 5:**
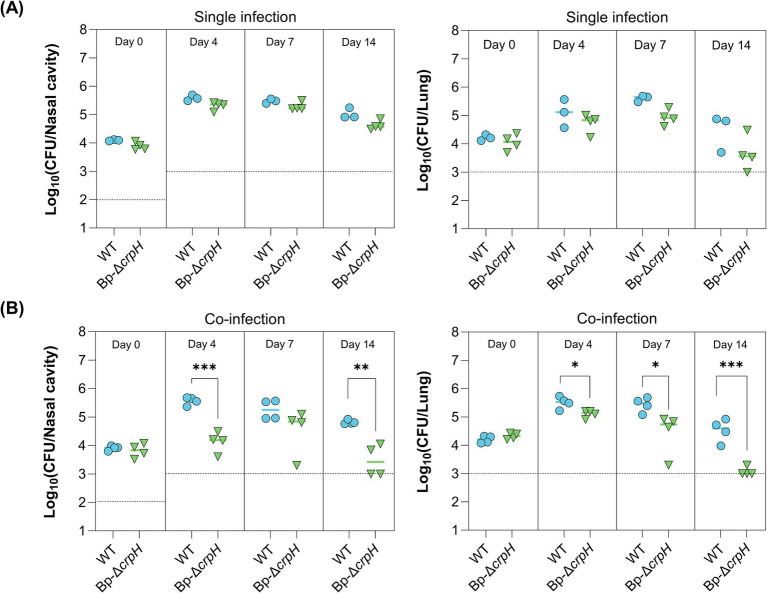
Role of CrpH in a murine infection model. **(A)** Bacterial loads were quantified in the nasal cavities (left panel) and lungs (right panel) following single infection with either WT *B. pertussis* or Bp-ΔcrpH. **(B)** Bacterial colonization was also assessed in the nasal cavities (left panel) and lungs (right panel) of mice co-infected with both strains. Each point represents an individual mouse, and the dashed lines indicate the limits of detection. Statistical significance was determined using a non-parametric permutation-based ANOVA followed by Cohen’s *d* test. In nasal co-infection, significant differences were observed at J4 (*p* = 0.000351, *d* = 5.12, 95% CI 4.03–16.7) and J14 (*p* = 0.00299, *d* = 3.40, 95% CI 2.77–39.7). In lung co-infection, significant differences were observed at J4 (*p* = 0.0198, *d* = 2.23, 95% CI 1.30–7.24), J7 (*p* = 0.0464, *d* = 1.77, 95% CI 1.39–7.92), and J14 (*p* = 0.000552, *d* = 4.71, 95% CI 3.37–17.4).

To evaluate the *in vivo* role of CrpH, we tested whether its inactivation influences the ability of the mutant strain to colonize the respiratory tract of mice. We infected mice with the WT or Bp-Δ*crpH* strains or co-infected them with equal numbers of both, and we determined the numbers of bacteria in the noses and lungs of the animals over time. The colonization profiles of the two strains did not significantly differ from one another when they were administered separately (Figure 5A). However, in co-infection experiments, Bp-Δ*crpH* exhibited a reduced capacity to persist and replicate compared to the WT parent, indicating that expression of this gene confers a competitive advantage *in vivo* (Figure 5B; Supplementary Figure S4). Thus, CrpH promotes the adaptation of *B. pertussis* to host conditions and to its replication and persistence in the respiratory tract, most likely because it indirectly contributes to respiration. Of note, similar results were obtained for the *crtA* mutant (Hachmi et al., 2026), consistent with the two genes being involved in the same pathway.

### CrpH is the prototype of a PepSY_TM subfamily

**Figure 6:**
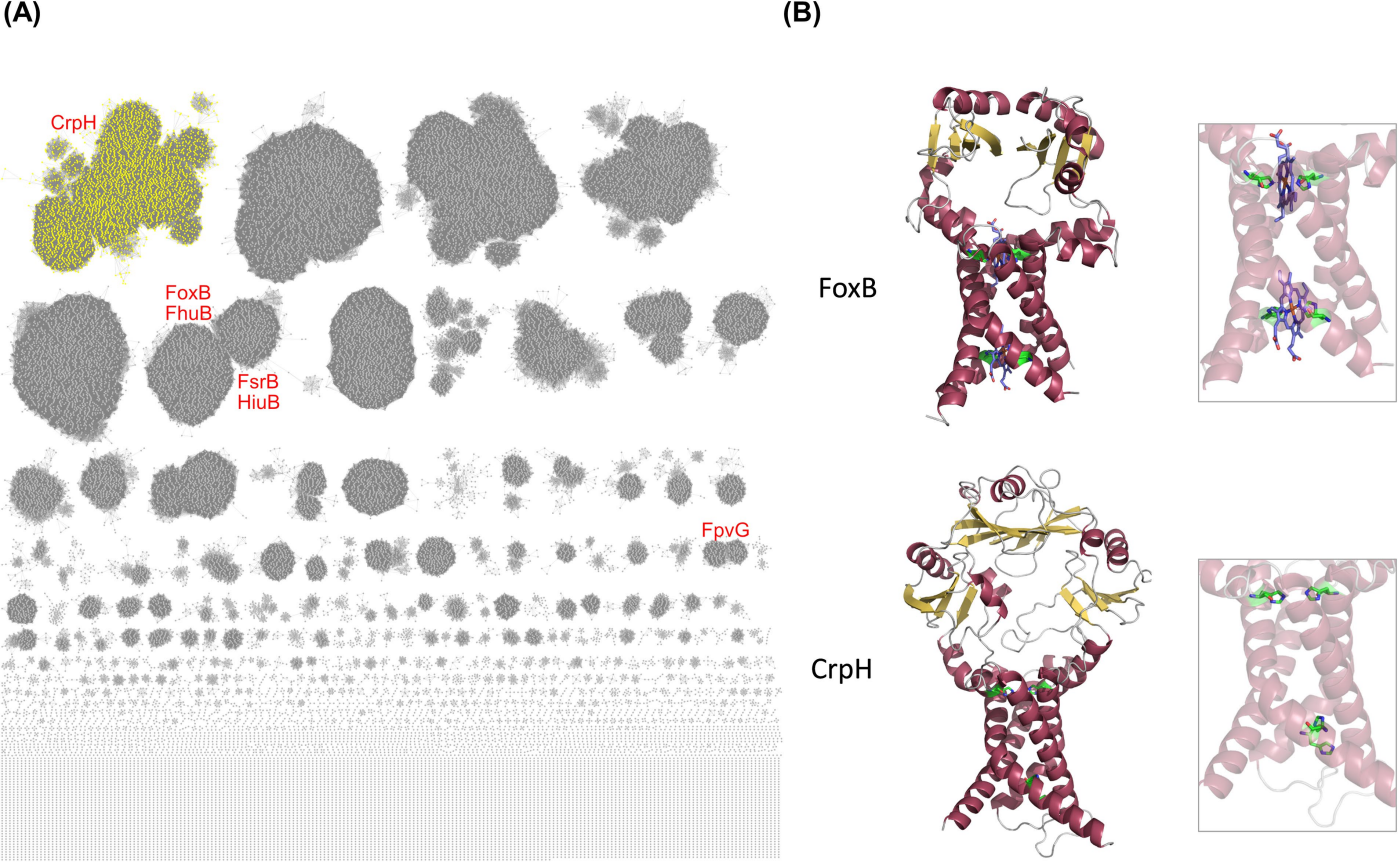
*In silico* analyses of the CrpH family. **(A)** Representative node network of the PepSY_TM superfamily (SSN performed with an AST value of 82; <80% sequence redundancy). CrpH belongs to the yellow cluster. Other known PepSY_TM proteins were positioned on this network. **(B)** X-ray structure of FoxB and AlphaFold model of CrpH. The structure of FoxB shows two heme groups axially each chelated by two conserved His residues. The hemes are involved in the transfer of an electron for the reduction of ferrioxamine bound in the periplasmic cavity. In the CrpH model the His residues are present at similar positions as in FoxB, two on each face of the membrane. The left panels show the entire proteins, while the right panels focus on the transmembrane regions. Note that some eukaryotic membrane-associated metal reductases (Supplementary Figure S2) have similarly positioned His residues, suggesting a conserved catalytic mechanism, although they have other family signatures.

To identify CrpH orthologs, we collected all proteins that respond to the PepSY_TM Pfam signature in the NCBI database (>195,000 proteins), reduced sequence redundancy to 80% (>41,000 proteins) and performed a sequence similarity network (SSN) analysis. This revealed that the PepSY_TM superfamily is extremely diverse. CrpH belongs to a large sequence cluster of >3,000 members distinct from those of FoxB and other PepSY_TM proteins, namely FhuB, FsrB, FpvG and HiuB (Hussein et al., 2024; Josts et al., 2021; Ong and O’Brian, 2023; Ganne et al., 2017; Hernandez-Ortiz et al., 2026) (Figure 6A). We generated AF3 models of CrpH and the latter four proteins, as well as of proteins of related families, VciB (Peng and Payne, 2017) and FrcB (Small and O'Brian, 2011) and compared them with the X-ray structure of FoxB (Figure 6B; Supplementary Figure S5). All proteins have at least four predicted TM segments and large periplasmic domains forming a vast cavity. His residues are found in CrpH at similar positions as the functionally essential heme-binding His residues of FoxB, suggesting a similar activity. Interestingly, several eukaryotic metal reductases have related architectures with heme groups in the membrane region for extra-cytoplasmic catalysis (Hassett and Kosman, 1995; Ohgami et al., 2006) (Supplementary Figure S5).

To further characterize the CrpH orthologs we collected the PepSY_TM proteins found in operons with CrtA^Bp^ orthologs (Hachmi et al., 2026), that all belong to the CrpH sequence cluster. We performed sequence alignment of these proteins and built a Weblogo (Supplementary Figure S6). Conservation among CrpH orthologs is mostly confined to the peri- and trans-membrane regions, indicating very diverse substrate-binding periplasmic cavities (Supplementary Figure S6).

To investigate if CrpH orthologs might be generally related to aerobic respiration, we analyzed >40,000 fully sequenced bacterial genomes for the co-occurrence of *crpH* orthologs with genes coding for copper-related proteins (Supplementary Table S3). The majority of bacteria encoding putative CrpH orthologs also encode HCO complexes and orthologs of ScoI, a copper chaperone involved in HCO assembly (Canonica et al., 2019), whereas the co-occurrence of *crpH* genes with genes coding for proteins involved in respiration on nitrogen species is much lower (Supplementary Table S4).

Collectively, our *in silico* analyses revealed that CrpH is distinct from previously reported bacterial PepSY_TM proteins. We identified thousands of putative orthologs in various bacteria. CrpH orthologs harbor invariant His residues like PepSY_TM-type ferrireductases, suggesting that they might have an analogous activity. They are found in the genomes of bacteria that all harbor one or several clusters of HCO-coding genes for aerobic respiration. Together with our wet-lab data, these elements suggest that these proteins might reduce copper to support HCO function. The periplasmic cavity of CrpH orthologs is poorly conserved, indicating that they are unlikely to all have the same substrates.

### Functional investigation of CrpH

We tried to produce CrpH as a recombinant protein in *Escherichia coli* to test its function *in vitro*. Unfortunately, all attempts were unsuccessful, irrespective of the expression system, culture medium or temperature. In a few instances a faint protein band at the expected size for CrpH was detected by immunoblotting (Supplementary Figure S7), but production could not be scaled up for extraction and purification. We also detected a faint protein band in *B. pertussis* extracts between 100 and 150 kDa, suggesting that CrpH forms a homodimer or a complex with another protein in the native host (Supplementary Figure S7). Overproduction could not be achieved in *B. pertussis* either. Thus, CrpH appears to be expressed at very low level and/or to be quickly degraded. It might be detrimental in excess, accounting for its tightly regulated expression.

**Figure 7:**
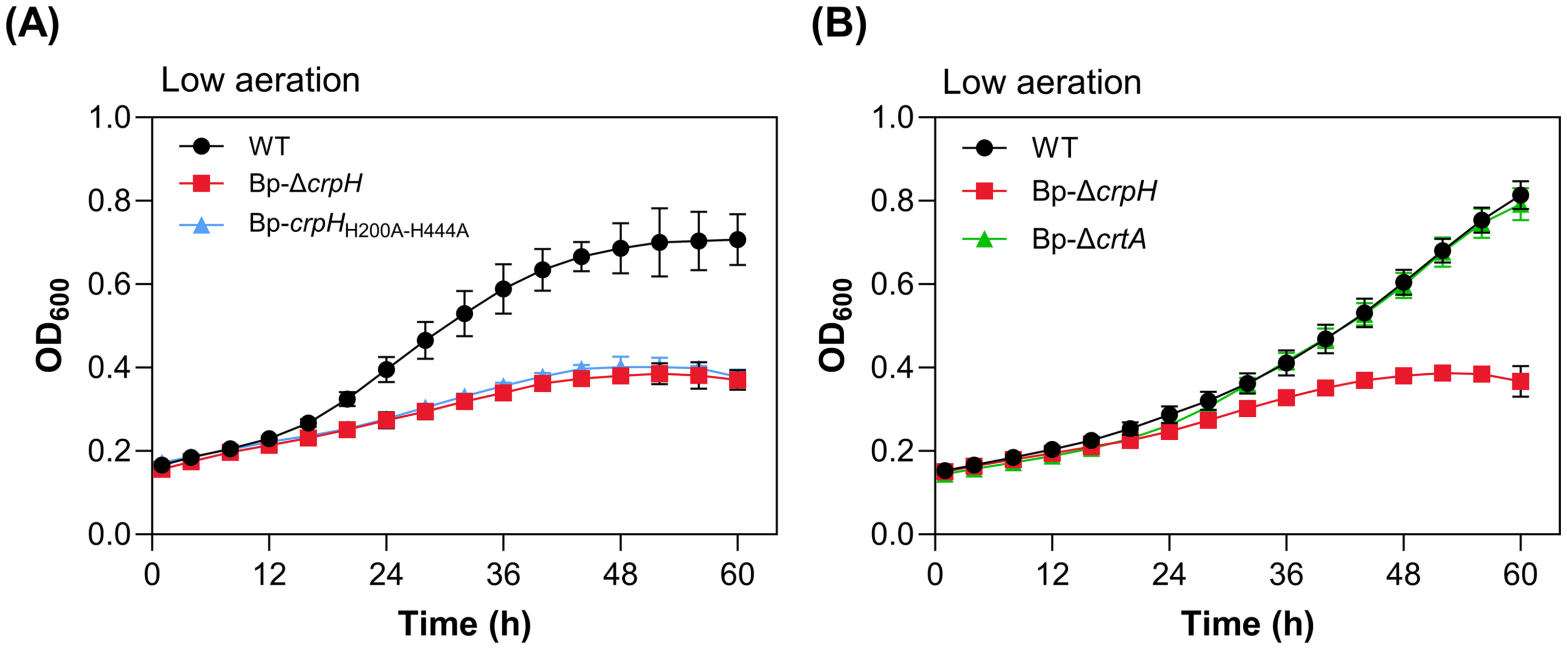
Functional characterization of CrpH. **(A)** Growth curves of WT, Bp-Δ*crpH*, and Bp-Δ*crpH*_H200A-H444A_ under low-aeration conditions in medium supplemented with 2 μM CuSO_4_. **(B)** WT, Bp-Δ*crpH*, and Bp-Δ*crtA* were grown under low-aeration conditions in medium supplemented with 500 μM Trien to induce copper limitation. The Bp-Δ*crtA*_KO_ strain does not phenocopy Bp-Δ*crpH*, indicating that CrpH does not depend on the Cu complex imported by CrtA.

We asked whether the invariant His residues of CrpH are required for its function. Those predicted to be on the periplasmic side of the transmembrane helices (Figure 6B) were both substituted with Ala residues. The effect of these replacements was evaluated by determining the growth phenotype of the recombinant strain, Bp-*crpH*_H200A-H444A_, compared to the WT and Bp-Δ*crpH* strains. Bp-*crpH*_H200A-H444A_ exhibited a similar growth defect as Bp-Δ*crpH*, supporting the functional importance of these His residues (Figure 7A). The mutant protein from *B. pertussis* membrane extracts migrated at the same place as its native counterpart, suggesting that it was correctly assembled (Supplementary Figure S7).

FoxB and its HiuB ortholog in *Caulobacter vibrioides* are encoded next to TBDTs that import ferrioxamine, which then serves as the substrate of these two enzymes. We reasoned that if similarly, the Cu-chelate complex imported by CrtA^Bp^ is the CrpH substrate, inactivation of either gene should have similar effects. Note that the copper complex(es) imported by CrtA *in vivo* have not been identified, but in laboratory conditions, CrtA^Bp^ transports Cu(II) chelated by small carboxylate-rich organic molecules (Hachmi et al., 2026). However, Bp-Δ*crtA* displayed a growth profile indistinguishable from that of the parental strain in low-aeration conditions, unlike Bp-Δ*crpH* (Figure 7B). This indicated a probable redundancy in Cu import (Hachmi et al., 2026) and also that CrpH can function independently of the CrtA-imported copper complex.

### Partial functional overlap of *crpH* and *ccoG*

**Figure 8:**
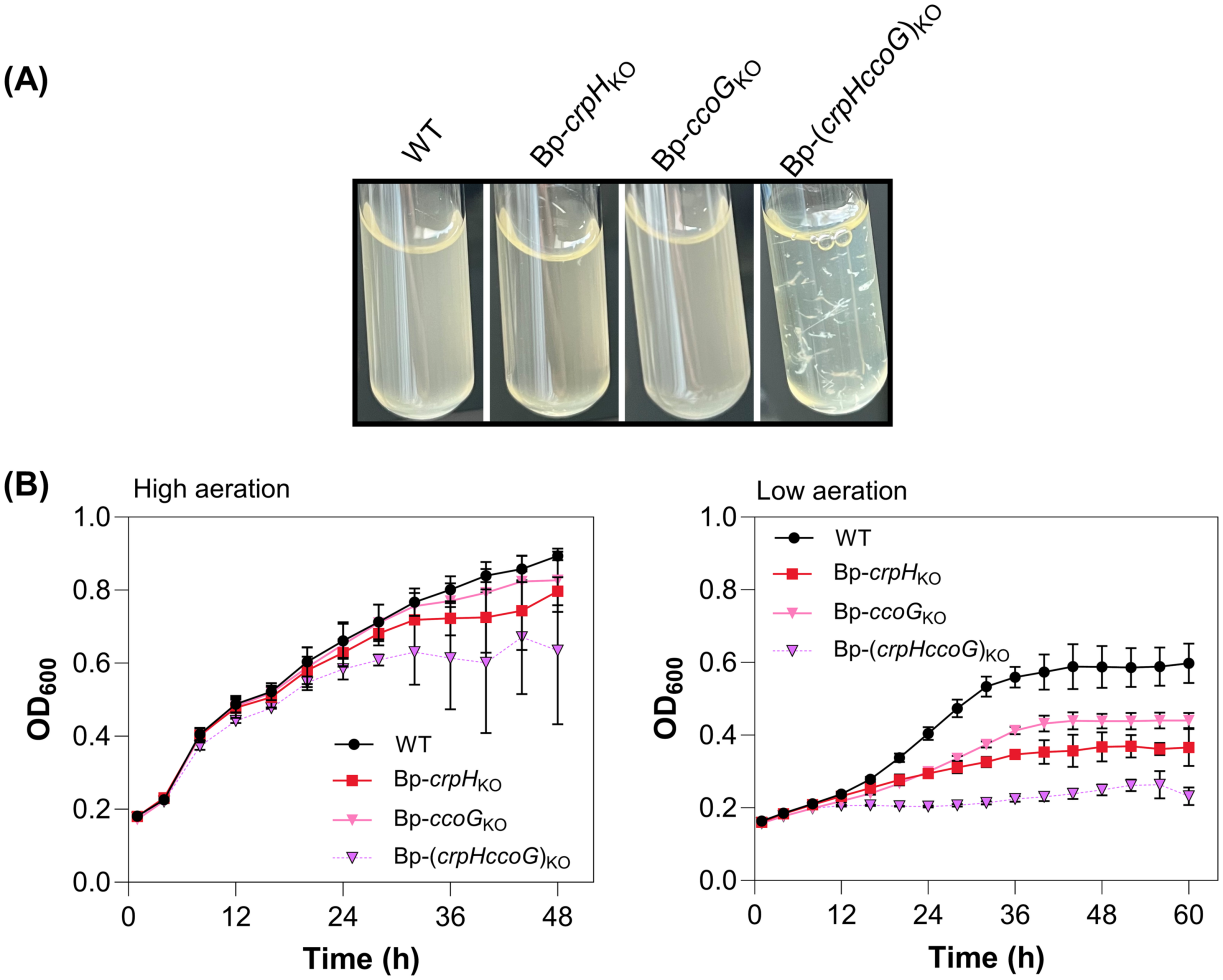
Effect of *ccoG* inactivation. *CcoG* is a putative cytoplasmic Cu(II) reductase. **(A)** Aggregation phenotype of Bp-(*crpHccoG*)_KO_ in high-aeration conditions in tubes. **(B)** WT, Bp-*crpH_KO_*, Bp-(*ccoG*)_KO_, and Bp-(*crpHccoG*)_KO_ were grown under high- (left panel) or low-aeration conditions (right panel) in 96-well plates in medium supplemented with 2 μM CuSO_4_. Data are representative of three biological replicates and correspond to the mean ± SD of three technical replicates.

To test in a different way the hypothesis that CrpH might be a copper reductase, we attempted to inactivate the gene coding for an unrelated putative copper reductase involved in HCO assembly in other bacteria. Thus, in *Rhodobacter capsulatus*, the membrane-associated protein CcoG reduces Cu(II) in the cytoplasm before Cu(I) export and delivery to periplasmic chaperones for incorporation into the HCO cbb3-Cox complex (Marckmann et al., 2019). CcoG orthologs are also found in non-cbb3-producing bacteria. In *B. pertussis* the *ccoG* ortholog, *bp2173*, is next to the *ccoNOQP* operon (*bp2169-72*) which codes for a non-functional cbb3-Cox, since *ccoN* is a pseudogene. Knocking *ccoG* out was easily achieved in the WT strain but extremely difficult in Bp-Δ*crpH*, with a single clone obtained after several attempts. The Bp-(*crpHccoG*)_KO_ strain showed a strong aggregation phenotype in aerated cultures performed in flasks or tubes (Figure 8A). Nevertheless, its growth was only mildly affected in high-aeration cultures in plates, in line with a major role of bd-Qox in these conditions (Figure 8B). In low-aeration conditions, however, Bp-(*crpHccoG*)_KO_ failed to grow even with copper supplementation, while the single *ccoG* mutant showed a milder growth defect (Figure 8B). This synthetic growth phenotype supports to notion that the two proteins represent parallel yet non-redundant pathways that support HCO-mediated respiration.

## Discussion

In this work we describe the prototype of a new subfamily of PepSY_TM proteins. Together with the copper regulation of the CrpH-coding operon in *B. pertussis* and the presence of *crtA*, a TonB-dependent copper transporter gene in the same operon (Hachmi et al., 2026; Roy et al., 2022), our data indicate that CrpH is related with copper, unlike other known PepSY_TM enzymes. *In silico* analyses showed that CrpH and its orthologs belong to a specific sequence cluster of PepSY_TM proteins and are found in various bacteria that all harbor HCO-coding operons. We speculate that it might be a copper reductase, but further investigations will be required to confirm this hypothesis.

Transition metals play dual roles at the host-pathogen interface (Murdoch and Skaar, 2022; Sheldon and Skaar, 2019), but the notion that mammals also use copper nutritional stress as an antimicrobial tool is rather recent (Besold et al., 2018; Buglino et al., 2025). For *B. pertussis*, the reduced competitiveness of the *crtA* mutant in mice suggests that this bacterium might similarly experience copper scarcity in the course of infection (Hachmi et al., 2026). The deployment of copper provision pathways for specific client proteins, such as HCOs in aerobic bacteria or methane monooxygenase in methanotrophs, may be an emerging theme in bacteria (Khalfaoui-Hassani et al., 2023; Khalfaoui-Hassani et al., 2018; Buglino et al., 2025; Dassama et al., 2016). In contrast, our *in vitro* results showed that CrpH is necessary for optimal growth in low aeration conditions even when copper is available, indicative of a role in copper utilization rather than acquisition. *B. pertussis* respires exclusively on molecular oxygen, and its HCO complexes, in particular bo-Qox, are important in murine models of infection (McKay et al., 2024; Gonyar et al., 2019). The role of CrpH for growth in the absence of the copper-independent respiratory oxidase bd-Qox, the decreased ATP levels of the *crpH* KO mutant, the reduced competitiveness of the mutant in mice and the transcriptomic data all support a role for CrpH in handling copper for HCOs in *B. pertussis*. Notably, expression of the *Pseudomonas aeruginosa* CprH ortholog (Uniprot Q9I682) is extinguished by the presence of endogenously produced hydrogen cyanide, an HCO poison, suggesting a similar link between CrpH and HCO complexes in that species (Frangipani et al., 2014).

Respiratory complexes widely vary between bacterial species depending on their physiology and their environment, and their assembly routes are also very diverse (Hederstedt, 2022; Llases et al., 2019). Insertion of Cu(I) and Cu(II) in the electron-accepting Cu_A_ center of aa3-Cox is mediated by the copper chaperones PCu_A_C and ScoI in several species, following reduction of the Cu_A_ site’s Cys residues by periplasmic thioredoxins (Canonica et al., 2019; Abicht et al., 2014; Abriata et al., 2008). Insertion of Cu(I) in the O_2_-reducing Cu_B_ center of aa3-Cox is mediated by other copper chaperones (Hederstedt, 2022). Copper insertion in the Cu_B_ site of cbb3-Cox in the model organism *R. capsulatus* successively involves Cu(II) import in the cytoplasm by the MFS transporter CcoA, reduction by CcoG, re-export of Cu(I) by the P_1B_-type ATPase CcoI, and finally action of periplasmic copper chaperones (Khalfaoui-Hassani et al., 2018; Marckmann et al., 2019; Trasnea et al., 2018). Little is known regarding the assembly of the Cu_B_ site in bo-Qox except that it involves an MFS copper importer in *E. coli* (Khalfaoui-Hassani et al., 2023). *B. pertussis* harbors genes coding for several of the above proteins, but their functions in the assembly of its HCO complexes have not been characterized.

The growth phenotypes of *crpH* mutants, the synthetic phenotype of the double *crpH-ccoG* mutant, the conserved metal reductase activity in the PepSY_TM family and the functional importance of CrpH’s invariant His residues lead us to speculate that CrpH might be a periplasmic copper reductase supporting HCO function. Several membrane-associated metal reductases of other Pfam families in eukaryotes and prokaryotes are functionally and structurally related to PepSY_TM proteins. They reduce Fe, and also Cu for some of them, at the extracytoplasmic side of the plasma membrane using heme groups for catalysis (Ohgami et al., 2006; Cain and Smith, 2021; Wyman et al., 2008). Thus, extracytoplasmic reduction of nutrient metals appears to be a widespread strategy.

CrpH comprises a large periplasmic cavity. In other PepSY_TM proteins, this cavity is used for substrate binding. However, CrpH activity appears to be independent of the copper complex imported by the co-regulated TBDT CrtA, and therefore its substrate(s) remains to be determined. The non-conserved periplasmic cavity among CrpH orthologs suggests the possibility of different partners across bacterial species. Of note, the reductase FsrB, which belongs to a different sequence cluster than CrpH, was shown to have a variety of Fe-siderophore substrates, suggesting that PepSY_TM proteins might not be highly selective (Ong and O’Brian, 2023).

The copper site(s) that rely on CrpH remain to be determined. The extremely severe growth phenotypes of the *crpHctaCDFGE* (aa3-Cox) and *crpHcyoABCD* (bo-Qox) mutants did not allow to draw firm conclusions, but they nevertheless indicated that both aa3-Cox and bo-Qox activities might be impacted by the absence of CrpH. However, the *crpH* mutant strain displayed aa3-Cox activity, suggesting that CrpH might have a minor role toward this complex. Transcriptomics analyses indicated that the absence of *crpH* affects *cyoABCD* expression. Of note, the latter genes are part of a large “copper locus” notably encompassing *cruR*, *crtA* and a bufferin operon (Leprevost et al., 2024). Altogether, thus, bo-Qox is a good client candidate for CrpH-mediated copper provision.

The bd-Qox and bo-Qox complexes of *B. pertussis* were predicted to have high- and low affinity for molecular oxygen, respectively. However, our results indicated that bd-Qox is important in high-aeration culture conditions. In some bacteria, bd-Qox provides protection from oxidative stressors likely more abundant in strongly aerated cultures, which would be consistent with our findings (Borisov et al., 2025; Xia et al., 2018). In contrast, the bo-Qox complex of *B. pertussis* was found here to be necessary in low-aeration conditions. Such as adaptation is not unprecedented, as the bo-Qox complex *Rhizobium etli* was reported to be utilized in low-oxygenation conditions (Lunak and Noel, 2015). Altogether thus, it is likely that the respiratory complexes have adapted to the specific niches of each species.

Unlike FoxB, CrpH could not be produced in recombinant form in *E. coli*, it was detected at very low level in *B. pertussis*, and attempts to overexpress *crpH* in *B. pertussis* were unsuccessful. We speculate that this may be related to its enzymatic function. The strict negative regulation of the operon by copper most likely evolved to limit the production of CrpH, possibly because of the danger of an imbalance to the periplasmic Cu(I)/Cu(II) ratio.

## Data Availability

The datasets presented in this study can be found in online repositories. The names of the repository/repositories and accession number(s) can be found in the article/Supplementary material.

## Footnotes

1
https://www.ebi.ac.uk/arrayexpress/

2
https://www.ebi.ac.uk/interpro/entry/pfam/PF03929

3
http://bioinformatics.org/cd-hit/

4
https://efi.igb.illinois.edu/efi-est/
