# Supplementary material for: CrpH of Bordetella pertussis, a prototypic PepSY_TM protein supporting heme-copper oxidoreductase function
Source: Front Microbiol. 2026 Mar 18;17:1786092. doi: 10.3389/fmicb.2026.1786092 (PMC13038864; doi:10.3389/fmicb.2026.1786092)
Supplement: Supplementary file 1 [file Data_Sheet_1.pdf]

Supplementary Material to

CrpH of *Bordetella pertussis*, a prototypic PEPSY_TM protein supporting heme-copper oxidoreductase function

Majda Hachmi, Gauthier Roy, Anne-Sophie Debrie, Stéphanie Slupek, Rudy Antoine, Françoise Jacob-Dubuisson

Includes 7 supplementary figures and 4 supplementary tables

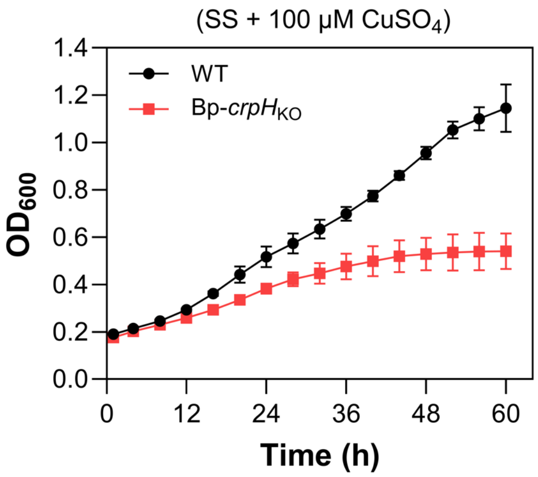

Suppl. Figure S1. Effect of copper on the growth phenotypes. The WT and Bp-*crpH*$_{KO}$ strains were grown under low-aeration conditions in medium supplemented with 100 µM $CuSO_4$.

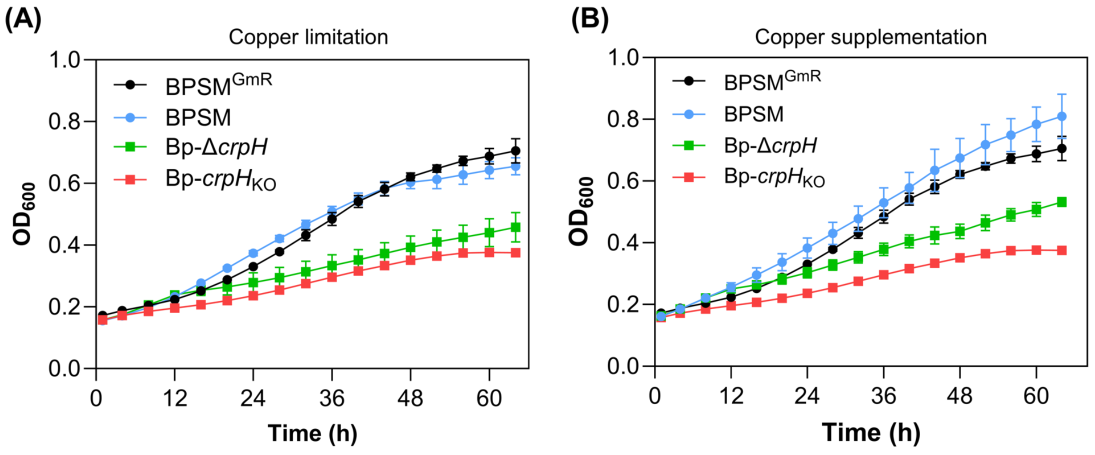

Suppl. Figure S2. Growth phenotypes of the deletion and insertion mutants. The WT, Bp-*ΔcrpH* and Bp-*crpH*$_{KO}$ strains were grown under low-aeration conditions in medium supplemented with 500 µM Trien (**A**) or 2 µM $CuSO_4$ (**B**). Note that two different strains of BPSM (unmarked and marked) were used to compare with the Bp-*ΔcrpH* (unmarked) and Bp-*crpH*$_{KO}$ (gentamycin resistance marker), respectively.

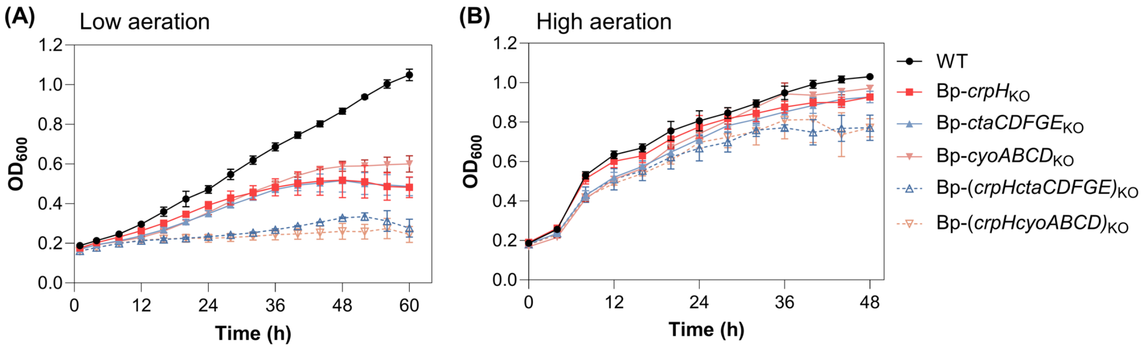

Suppl. Figure S3. Growth phenotypes of respiration mutants. The WT, Bp-*crpH*KO, Bp-*cyoABCD*KO, Bp-*ctaCDFGE*KO*,* Bp-(*crpHcyoABCD)*KO, and Bp-(*crpHctaCDFG*E)KO strains were grown under low- **(A)** or high-aeration **(B)** conditions in medium supplemented with 2 µM $CuSO_4$. The defective growth of Bp-*cyoABCD*KO and Bp-*ctaCDFGE*KO in **(A)** suggests that both complexes contribute to respiration in low-aeration conditions. Representative growth curves from three independent biological replicates are shown. The data represent the means ± SD of three technical replicates.

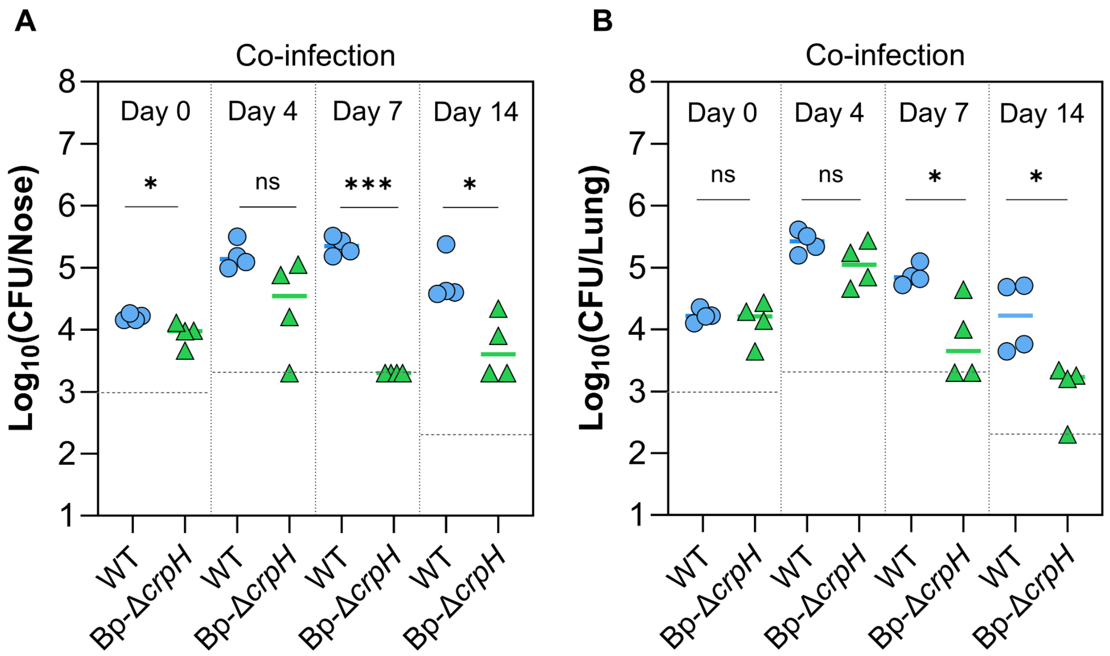

Suppl. Figure S4. Role of CrpH in a murine infection model. A second-infection experiment was performed to confirm the results shown in Fig. 4B. Bacterial colonization was assessed in the nasal cavities (left panel) and lungs (right panel) of mice co-infected with both strains. Each point represents an individual mouse, and the dashed lines indicate the limits of detection. Statistical significance was determined using a non-parametric permutation-based ANOVA (*, $p<0.05$; **, $p<0.01$; ***, $p<0.001$).

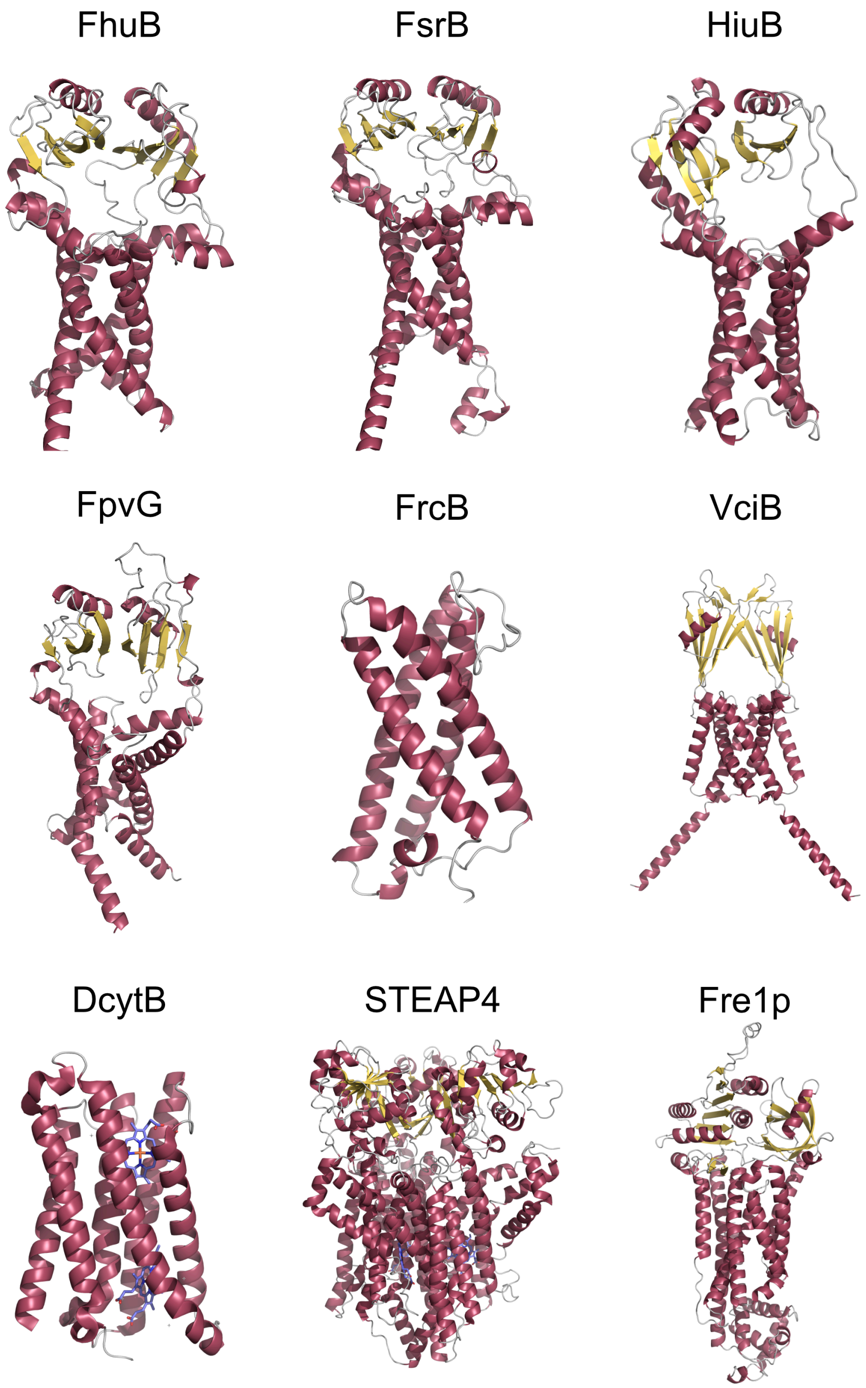

Suppl. Figure S5. Models of selected prokaryotic and eukaryotic metal reductases. AlphaFold models of previously reported PEPSY_TM proteins and related metal reductases, VciB (Pfam PEPSY_TM_like 2) and FrcB (Pfam Ni_Hyd_CYTB). VciB forms homodimers. The X-ray structures or AlphaFold models of related eukaryotic metal reductases are also represented, including human DcytB (PDB 5ZLE), human Steap4 (PDB6HD1) and Fre1P of *Saccharomyce*s. Heme groups (shown in blue) are found in the transmembrane regions of DcytB and Steap4.

A

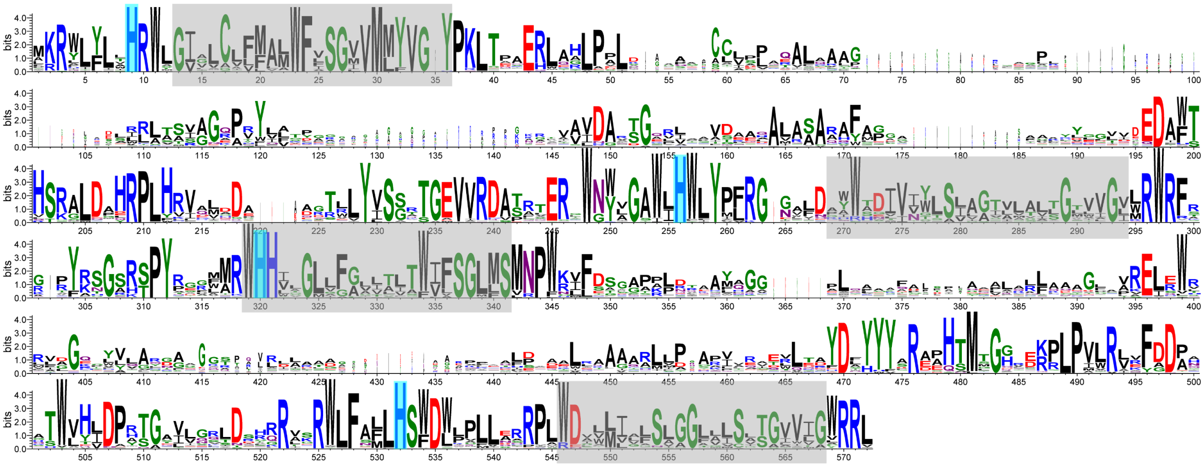

B

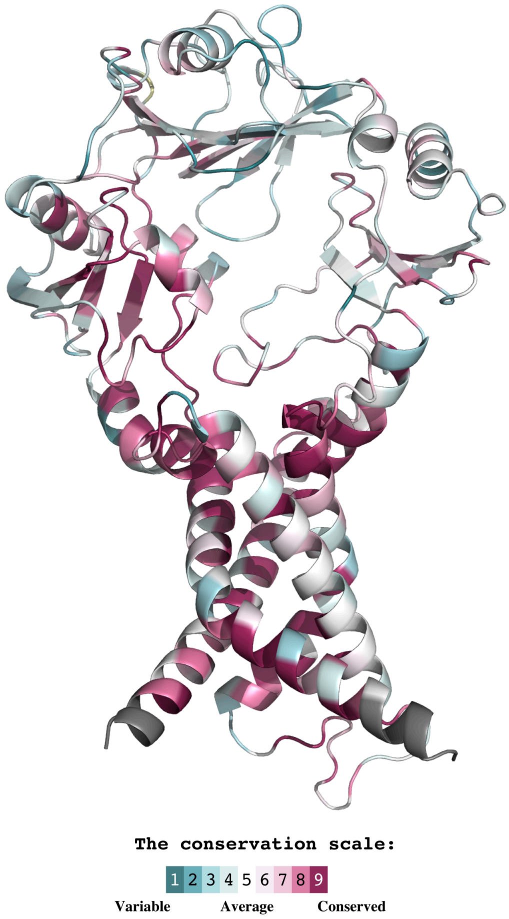

Suppl. Figure S6. Weblogo and conservation in the CrpH subfamily. In (A), The transmembrane segments and the four invariant His residues are highlighted in grey and cyan, respectively. In (**B**), the degree of conservation was plotted on the CrpH structural model. The figure was built using Consurf.

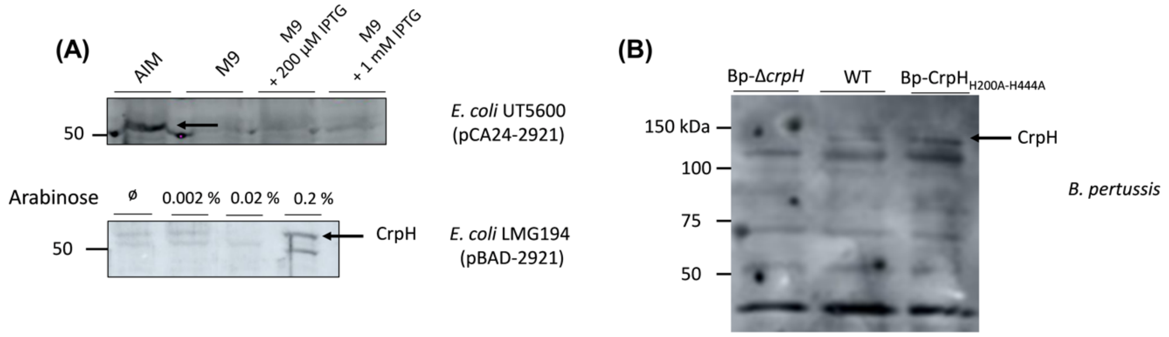

Suppl. Figure S7. Analysis of recombinant and native CrpH expression. (A), *E. coli* UT5600 and LMG194 were transformed with plasmids pCA24-2921 and pBAD-2921, respectively, that both carry *crpH*. Protein production was induced either naturally in AIM (Auto-Induction Medium) or by addition of various concentrations of IPTG or arabinose as indicated. A faint band was detected slightly above the 50-kDa marker that might correspond to CrpH (calculated size 54 kDa). CrpH was detected in membrane extracts using an anti-His antibody. **(B),** Membrane extracts of the WT, $Bp\text{-}crpH_{KO}$ and Bp-$\mathrm{CrpH}_{\mathrm{H200A\text{-}H444A}}$ strains were probed with polyclonal antibodies raised against synthetic peptides of CrpH. A faint band absent from the negative control was detected between 100 and 150 kDa in WT and Bp-$\mathrm{CrpH}_{\mathrm{H200A\text{-}H444A}}$, but not around 54 kDa. Several aspecific bands were present in the three extracts, most likely because of the poor quality of the antibody and the low levels of the protein of interest.

Table S1. Strains, plasmids, and primers used in this study (Excel file).

Table S2. RNAseq data (Excel file). Three biological replicates were obtained for BPSM and Bp-*crpH*$_{KO}$ grown under agitation (denoted High) or in static cultures (denoted Low). The cultures were performed in SS medium containing 20 mM $MgSO_4$, as these conditions were found to maximize expression of the operon. The data were deposited in ArrayExpress (https://www.ebi.ac.uk/arrayexpress/), under the accession number E-MTAB-16727.

Table S3. Analyses of assembled bacterial genomes (Excel file). The producing organisms and the presence of genes of interest in these bacteria are provided.

Table S4. Co-occurrences of CrpH orthologs with copper-related proteins in assembled bacterial genomes.

	CrpH_PepSY_TM
SCO1-SenC	98,6%
COX1	97,4%
Cyt_bd_oxida_I	97,0%
Cyt_bd_oxida_II	96,9%
Cu-oxidase_4	95,3%
COX2	94,4%
Cu-oxidase_2	94,2%
Cu-oxidase_3	94,1%
Cu-oxidase	93,6%
CyoB	89,0%
Cytochrome_CBB3	80,8%
PCuAC	74,8%
Copper-bind	74,2%
FixO	64,5%
Sod_Cu	63,8%
FixG_C	62,7%
CtaG_Cox11	53,0%
NnrS	52,1%
CusF_Ec	42,7%
DUF2282	33,7%
Caa3_CtaG	24,8%
NosL	21,6%
nos_propeller	20,5%
